# Editorial: Impact of Climate Change on Immune Responses in Agricultural Animals

**DOI:** 10.3389/fvets.2021.732203

**Published:** 2021-07-23

**Authors:** Mariangela Caroprese, Barry J. Bradford, Robert P. Rhoads

**Affiliations:** ^1^Department of Agriculture, Food, Natural Resources, and Engineering, University of Foggia, Foggia, Italy; ^2^Department of Animal Science, Michigan State University, East Lansing, MI, United States; ^3^Department of Animal and Poultry Sciences, Virginia Tech, Blacksburg, VA, United States

**Keywords:** climate change, immune response, farm animals, dairy cow, camel (*Camelus dromedaries*), broiler-chicken, feedlot beef cattle

Livestock production and welfare currently face critical challenges from climate change and global warming that will continue in the future with the predicted increase in the frequency and intensity of heat waves. Filipe et al. aptly described climate change as a very complex problem existing at a global level. Climate change contributes to the impairment of animal welfare and health both by the spread of vector-borne diseases and by its impact on the immune system of the animals, inducing immune suppression and increasing the susceptibility of animals to infections. In livestock, high-yielding animals, characterized by an accelerated metabolism and genetic selection based on production level in near-optimal conditions, may be the most endangered by climate change and the least resilient (1, 2). Based on this, the impact of climate change on production and reproduction of livestock will cause important economic losses (Filipe et al.). The genetic selection of livestock to address immune challenges in the face of more frequent heat stress can be an opportunity. As a result, Filipe et al. suggests that the valorisation of autochthonous livestock breeds known to be highly resilient and disease resistant, to have low dietary needs (acceptable production levels despite frugal rations) and to produce high quality products could be a strategy for future livestock management.

Alterations in immune responses are observed in many species under heat stress; in broiler chickens, a particular sensitivity of lymphoid tissues to heat stress has been observed, with a depression of antigen-specific humoral immune responses, a substantial decrease in the number of splenic lymphocytes, and a decrease in immature B cells in bursal follicles. Hirakawa et al. suggest that the alteration in immune responses measured in broiler chicken under heat stress can be related to reduced feed intake and to insufficient macro-energy sources and/or micro-nutrients delivered to the lymphoid tissues, resulting in the incomplete proliferation and differentiation of T and B cells. As a matter of fact, the maintenance of tissue-specific homeostasis requires sufficient amounts of nutrients (e.g., glucose, fatty acids, amino acids, minerals, and vitamins) to meet the specific tissue requirements. The insufficient nutritive supply to primary and secondary lymphoid tissue induced by heat stress may cause pathological atrophy of tissues, reducing lymphocyte number and degrading functional structures.

However, one of the species most negatively affected by heat stress is cattle, and particularly dairy cows during their entire life cycle, as documented in the extensive review by Dahl et al.. Heat stress impacts cows starting from gestation, when elevated maternal temperatures can be detrimental to embryonic and fetal development by altering cell proliferation, migration, differentiation, and programmed cell death. These effects are probably attributed to altered nutrient partitioning responsible for restricting the ontogeny of the fetal immune system. Maternal hyperthermia during early stages of fetal development may impact the differentiation, migration, and/or establishment of the highly proliferative pool of lymphoid-hematopoietic progenitor cells that are destined to become immune cells. In addition, the postnatal period is critical for the passive transfer of immunity; prenatal heat stress impairs passive transfer of colostral IgG. Newborn calves from heat-stressed dams, fed colostrum from their respective mothers, have reduced apparent efficiency of IgG absorption, compared with calves born to cooled dams and receiving their dam's colostrum, thus compromising the immune function of offspring from birth through weaning (3). Heifers also suffer from hyperthermia as their ability to dissipate metabolic heat gradually decreases, resulting in reduced heat tolerance during heifer development. Negative impacts of heat stress on lactational performance are well-known, but heat stress also has substantial impacts on a mature dairy cow's immune function and health, as suggested by the association of summer season with increased disease incidence. Finally, recent studies provide solid evidence that during the dry period, heat stress has significant negative effects on innate and adaptive immune function of both the dam and the offspring, influencing the morbidity and mortality of early lactation cows and calves from birth to first calving.

Heat abatement by physical modification of the environment is an effective and profitable strategy to manage heat stress in dairy cattle leading to successful outcomes that minimize the negative impacts of heat stress on animal health. Although heat abatement for mature dairy cows, especially those housed in barns, has been extensively studied, additional effort is needed to develop effective and affordable cooling strategies for grazing dairy cattle, preweaned calves, and growing heifers to maintain immune function and optimal performance of these animals under field conditions.

In a retrospective study, Broadway et al. found no correlation between summer temperature patterns and cattle morbidity/mortality in feedlots in the Texas Panhandle. However, some of the negative effects of heat stress observed in a controlled study appeared to be mitigated by prophylactic yeast and yeast cell wall supplementation in nearly finished beef cattle. Heifers supplemented with a combined live yeast and yeast cell wall product had decreased vaginal temperature and respiration rate and increased water intake in comparison to non-supplemented control heifers (Broadway et al.).

In contrast to cattle, the dromedary camel can be considered a model of different immune reactivity to heat stress, thus helping to better understand thermotolerance mechanisms developed in livestock adapted to hot climates. In his manuscript, Hussen shows enhanced phagocytosis and ROS production activities of heat-challenged camel neutrophils and monocytes toward *S. aureus*, which is in contrast to the reported inhibitory effect of heat stress on antimicrobial functions of bovine neutrophils. Camel lymphocytes demonstrate higher resistance to heat stress compared to granulocytes and monocytes.

Collectively, these contributions highlight the importance of considering animal resilience in the face of heat stress when designing breeding programs, feeding strategies, and environmental modifications for livestock. These strategies will be critical to the ongoing sustainability of animal agriculture as we adapt to a changing climate in the twenty-first century.

## Author Contributions

MC wrote the first draft of the manuscript. BB and RR contributed to manuscript revision, read, and approved the submitted version. All authors contributed to the article and approved the submitted version.

## Conflict of Interest

The authors declare that the research was conducted in the absence of any commercial or financial relationships that could be construed as a potential conflict of interest.

## Publisher's Note

All claims expressed in this article are solely those of the authors and do not necessarily represent those of their affiliated organizations, or those of the publisher, the editors and the reviewers. Any product that may be evaluated in this article, or claim that may be made by its manufacturer, is not guaranteed or endorsed by the publisher.
